# The enhancement of immunoactivity induced by immunogenic cell death through serine/threonine kinase 10 inhibition: a potential therapeutic strategy

**DOI:** 10.3389/fimmu.2024.1451796

**Published:** 2024-11-01

**Authors:** Xiaoli Xia, Yixin Wang, Minghui Wang, Jian Lin, Ruiheng Wang, Shufeng Xie, Yaoyifu Yu, Jinlan Long, Zixuan Huang, Huajian Xian, Wenjie Zhang, Chaoqun Lu, Wenfang Wang, Han Liu

**Affiliations:** Shanghai Institute of Hematology, State Key Laboratory of Medical Genomics, National Research Center for Translational Medicine at Shanghai, Rui Jin Hospital, School of Medicine and School of Life Sciences and Biotechnology, Shanghai Jiao Tong University, Shanghai, China

**Keywords:** ICD-high subtype, ICD-low subtype, STK10, ICD inducer, prognosis

## Abstract

**Introduction:**

Immunogenic cell death (ICD) is capable of activating the anti-tumor immune response of the organism; however, it is concurrently a complex process involving multiple factors. The specific factors that impact the occurrence of ICD remain undefined.

**Methods:**

Through cluster analysis, patient specimens retrieved from the TARGET, TCGA, and GEO AML databases were categorized into two subtypes based on the expression levels of ICD-related genes: ICD-high and ICD-low. We compared the prognostic survival outcomes, pathway enrichment analysis, and immune cell infiltration between these two subtypes. Additionally, we identified factors related to AML development from multiple databases and verified the role of these factors both in vivo and in vitro in activating the immune response during the occurrence of ICD.

**Results and discussion:**

In the ICD-high subtype, there was a notable increase in the abundance of immune cell populations, along with the enrichment of pathways pertinent to the activation of various immune cells. Despite these immunological enhancements, this subgroup demonstrated a poorer prognosis. This phenomenon was consistently observed across various additional AML datasets, leading us to hypothesize that elevated expression of ICD genes does not invariably correlate with a favorable prognosis. Notably, STK10 exhibited elevated expression in AML, was associated with a poor prognosis, and showed synchronous expression patterns with ICD genes. Inhibition of STK10 led to the activation of ICD and the induction of an antitumor response. Moreover, when combined with other ICD inducers, it produced a synergistic anti-tumor effect. Our results reveal the impact of STK10 on ICD and underscore its key role in initiating ICD.

## Introduction

Immunogenic cell death, a regulated form of cell death (RCD), is essential in tumor immunotherapy (1). Recent research has focused on investigating the mechanisms of ICD, with particular attention to the release of ATP, exposure of calreticulin (CALR), and secretion of the nuclear protein HMGB1 (2). These factors are crucial in activating the adaptive immune system, as ATP attracts dendritic cell precursors expressing purinergic receptors (3) and calreticulin signals for the phagocytosis of dying cancer cells (4). Additionally, HMGB1 activates toll-like receptor 4 (TLR4), promoting the maturation of dendritic cell. Preclinical trials have affirmed chemotherapeutic agents, such as doxorubicin, mitoxantrone, oxaliplatin, bortezomib, cyclophosphamide, and anthracycline can trigger ICD, thereby activating anticancer immune responses (5). In 2020, two anti-cancer drugs based on the concept of ICD, belantamab mafodotin (6) and lurbinoctedin (7), were approved by the FDA.

Acute myeloid leukemia (AML) is characterized by the malignant transformation of myeloid progenitor cells, which are arrested in a premature and inherently proliferative condition (8). The primary treatment approach for AML is chemotherapy, and enhancing the induction of ICD has the potential to enhance the efficacy of chemotherapeutic agents in both experimental models of AML and individuals (9, 10). Previous studies have demonstrated that blocking the protein phosphatase 1/GADD34 complex can mimic the CALR translocation triggered by anthracyclines. Anderson’s research suggests that bortezomib enhances the immunogenicity of multiple myeloma cells by activating the cGAS/STING pathway and promoting the production of type I interferons. Furthermore, studies have demonstrated that STING agonists significantly enhance bortezomib-induced ICD (11). However, the precise role of ICD in AML remains incompletely elucidated. Further investigation is warranted to elucidate the clinical significance of ICD biomarkers in AML patients.

Serine/threonine kinase 10 (STK10), a member of the serine/threonine kinase family, plays an important role in various biological processes (12). STK10 features an N-terminal kinase domain and a C-terminal phosphorylation domain, allowing it to phosphorylate serine/threonine residues. Its primary role lies in the regulation of the cell cycle, signal transduction, and cell apoptosis. In the context of tumorigenesis, aberrant expression of STK10 can impact tumor cell proliferation, differentiation, and apoptosis, thereby influencing the initiation and progression of tumors. These findings are supported by evidence showing that STK10 induces PLK1 activation in COS-7 cells, and that upregulation of a kinase-inactive STK10 in 3T3 fibroblasts impedes cell proliferation (12). Nevertheless, the function of STK10 in modulating apoptosis is subject to debate. Loss-of-function mutations lead to reduced apoptotic activity (13), whereas silencing STK10 with siRNA leads to heightened cell death in Ewing’s sarcoma cell lines (14). These observations imply that the impact of STK10 is contingent upon the cell type and environmental conditions.

The precise role of STK10 in the promotion of ICD is not yet fully understood. Our research has unveiled that the activation of ICD-related genes triggers comparable immune responses in various tumor models. Additionally, we have identified two subtypes linked to ICD in individuals with acute myeloid leukemia (AML) through consensus clustering. Of particular importance, the ICD-high subtype is characterized by an augmented infiltration of immune cells and heightened immune response signals, yet it is associated with inferior clinical outcomes compared to the ICD-low subtype. These findings suggest that the induction of an immune response through ICD is a multifaceted process influenced by multiple factors, which can counteract the favorable prognosis typically associated with ICD and lead to markedly reduced survival rates.

Furthermore, not all ICD-associated biomarkers are associated with a good prognosis. The relationship between STK10 and ICD-associated biomarkers is significant, as evidenced by their influence on the expression of these biomarkers following cell death induced by STK10 inhibitor. Our collective goal is to identify novel targets for the treatment of AML and provide additional guidance for clinical management.

## Materials and methods

### Data availability

The TARGET mRNA expression data of 303 normal and 482 AML patients, along with their corresponding clinicopathological data, were obtained from the GDC Data Portal (https://portal.gdc.cancer.gov). The TARGET RNA-seq alignment and count files were generated using GENCODE v36 gene annotation (https://gdc.cancer.gov/about-data/gdc-data-processing/gdc-reference-files). To eliminate data dimensionality, the TPM matrix of TARGET was standardized by applying a log2 transformation to the TPM values plus one. Adding one to each TPM value prevents issues with logarithmic transformation when dealing with zero TPM values. The TCGA AML and GTEX FPKM datasets were acquired from UCSC Xena Portal (http://xena.ucsc.edu). Subsequently, we converted FPKM values into TPM values using the formula: TPMi = FPKMi × 1000000/(FPKM0+⋯.+FPKMm). In the formula, i represents gene i, while m denotes the total number of genes. Finally, the resulting TPM values were normalized through a log2(TPM + 1) transformation. We extracted the OHSU (15) AML dataset from cBioPortal (https://www.cbioportal.org/datasets).

Additionally, we obtained expression and clinical information from Gene Expression Omnibus (GEO) datasets (https://www.ncbi.nlm.nih.gov), which consisted of GSE71014 (n = 104) (16), GSE16432 (n = 420) (17), GSE14468 (n = 244) (18) and GSE76009 (n = 227) (19). Samples lacking complete prognostic information were excluded, and probes without corresponding gene symbols were also eliminated from further analysis. Both GSE71014 and GSE76009 were acquired using the GPL10558 detection platform, while different GEO platforms (GPL), including GPL8650-8654 and GPL10105-10108, contributed to the acquisition of GSE16432. The acquisition of GSE14468 was performed with GPL570, followed by normalization of raw data using Affymetrix Microarray Suite 5 (MAS5) to target intensity values at 100. Intensity values underwent log2 transformation and mean centering per probe set.

### Cluster analysis

Cluster analysis was conducted and presented using the SangerBox software (http://www.sangerbox.com/tool) (20), an online platform for data analysis. In brief, ConsensusClusterPlus (21) was employed for cluster analysis, utilizing agglomerative k-means clustering with a 1-pearson correlation distance and resampling 80% of the samples for 10 repetitions. Based on the assessment of the area under the cumulative distribution function (CDF) curve, an increase in K results in a gradual augmentation of the area beneath the CDF curve. To maximize this area while maintaining its magnitude, it is advisable to minimize the decline rate of Delta based on evaluating CDF Delta’s downward trend. The optimal number of clusters was determined by considering both intra-group consistency and average agreement within each cluster group.

### Generation of Kaplan–Meier plots

Based on the expression level of the ICD gene, patient samples from various AML datasets were classified into two subtypes: C1 and C2. Survival analysis was conducted based on the survival time and living status of these samples. Kaplan-Meier (KM) plots were generated for survival analysis using sangerbox online platform, which is based on the R survival package. The final prognostic KM plots were presented with a hazard ratio (HR), a 95% confidence interval (CI), and a log-rank P value. A statistically significant result was considered when the P value was less than 0.05.

### Differential gene analysis

The TARGET AML samples were stratified into C1 and C2 subtypes through cluster analysis, followed by differential gene analysis using the limma method. Limma (linear models for microarray data, DOI:10.1093/nar/gkv007) is a statistical approach based on generalized linear models for identifying differentially expressed genes. In this study, we employed the R software package limma (version 3.40.6) to perform differential analysis and identify genes that exhibit significant differences between various comparison groups and control groups. Specifically, the expression profile dataset was subjected to multiple linear regression using the lmFit function. This was followed by the calculation of moderated t-statistics, moderated F-statistics, and log-odds of differential expression using eBayes function, which applies empirical Bayes moderation to standard errors. Finally, the significance of differential expression for each gene was determined. Volcano plots and heatmaps were generated using the differentially expressed genes in the R packages ggplot2.

### Gene Set Enrichment Analysis

The Gene Set Enrichment Analysis (GSEA) software (version 3.0) was obtained from the Broad Institute via the following link: http://software.broadinstitute.org/gsea/index.jsp.

The TARGET AML samples were classified into C1 and C2 subtypes based on cluster analysis, and we downloaded the c7.All.V7.4.Symbols.gmt and c2.cp.kegg.v7.4.symbols.gmt collections from the Molecular Signatures Database for KEGG pathway and immunologic signature analysis (http://www.gseamsigdb.org/gsea/downloads.jsp). To evaluate relevant pathways and molecular mechanisms, gene expression profiles were utilized in conjunction with phenotype grouping, using a minimum gene set of 5 and a maximum gene set of 5000, in addition to performing 1000 resamplings. The ranked list was generated through differential gene analysis, where the p-value is defined as follows: The absolute value of the p-value equals −log2(adjusted p-value), with the adjusted p-value calculated by limma from differential gene analysis.

### GO annotation

For the functional enrichment analysis of gene sets, we utilized the GO annotation from the R software package org.Hs.eg.db (version 3.1.0) as the background to map genes into the reference set. Enrichment analysis was conducted using clusterProfiler (version 3.14.3), an R software package, to obtain gene sets enrichment results. The minimum gene set size was defined as 5, while the maximum gene set size was limited to 5000. Statistical significance was determined by p < 0.05.

### Immune cell infiltration estimations

The estimation of immune cell infiltration was performed using the IOBR R package (22) on the SangerBox online platform. IOBR provides a comprehensive resource for the systematic analysis of immune cell infiltrates in various cancer types, offering multiple immune deconvolution methods such as quanTIseq, TIMER, CIBERSORT, xCell, MCP-counter and EPIC algorithms. We utilized TARGET, GSE71014, and GSE76009 profiles for this analysis. Upon uploading the input file, IOBR’s estimation component automatically executes immune infiltration estimation using CIBERSORT. The resulting values encompass overall estimates of immune cell infiltration as well as specific levels and proportions of eight common immune cells infiltrating each sample. The proportions of these eight common immune cells are visualized using GraphPad Prism 6 software.

### Cell culture conditions and reagents

The leukemia cell lines MV411, THP1, NOMO1, and MOLM13 were obtained from American Type Culture Collection (ATCC, Rockefeller, MD, USA) and maintained at 37 °C in 5% CO_2_ in RPMI 1640 medium containing 10% (v/v) fetal bovine serum (FBS) and a 5% (v/v) mixture of penicillin and streptomycin. Bortezomib (S1013, Selleck) and SB633825 (HY-108333, MedChemExpress) were reconstituted according to the instructions. Samples were collected with written consent from the patients, and data analysis protocols were approved by the Ruijin Hospital Ethics Committee, adhering to the principles of the Helsinki Declaration.

### Western blot analysis and fractionation

For the preparation of whole-cell extracts, cell pellets were washed with DPBS, resuspended in protein lysate containing 2% SDS, and boiled. Lysates were separated on sodium dodecyl sulfate-polyacrylamide gel electrophoresis, transferred to nitrocellulose membranes, and subsequently analyzed by immunoblotting. The antibodies used included STK10 (25471-1-AP; Proteintech), phospho-IF2α (44-728G; Thermo Fisher Scientific) and β-actin (3700; Cell Signaling Technology).

### Quantification of relative cell viability by CCK8

To evaluate the impact of Bortezomib and SB633825 on AML tumor cells, MV411, MOLM13, NOMO1, or THP1 cells were seeded into 96-well plates at a concentration of 1 × 10^4^ cells per well in 100 μL of growth medium. Cells were treated with different concentrations of Bortezomib, either alone or in combination with SB633825, at 37°C for 60 hours. Then, 10 μL of Cell Counting Kit 8 (CCK-8) reagent was added to each well, followed by incubation at 37°C for 1.5 h. Cell viability was measured at 450 nm using a microplate reader. Each experiment was repeated in triplicate, and statistical analysis was performed using GraphPad Prism 6 software. To calculate the cell survival rate, use the following formula: Survival rate (%) = (OD_experiment_ - OD_blank_)/(OD_control_ - OD_blank_) × 100%.

### Cell cycle and apoptosis analysis

The cell cycle analysis was conducted using the APC BrdU flow kit (552598, BD Biosciences). Cells were treated with 10 μM BrdU for 1 hour, followed by harvesting, fixation, permeabilization, and subsequent fixation before incubation with DNase to expose the incorporated BrdU. Subsequently, cells were stained with anti-BrdU-APC for 20 minutes at room temperature. Finally, cells were washed and resuspended in 7-AAD staining buffer. The analysis of stained cells was performed on a flow cytometer.

For the Annexin V apoptosis assay, APC Annexin V Apoptosis Detection Kit (559763, BD Biosciences) was used. The APC annexin V apoptosis detection was performed using flow cytometry in accordance with the manufacturer’s protocol, and the data were processed using FlowJo. Early apoptotic cells were identified as those that were APC Annexin V-positive and 7-AAD-negative. Cells that stained positive for both APC Annexin V and 7-AAD were either in the end stage of apoptosis or undergoing necrosis, while cells that stained negative for both dyes were considered alive.

### Depletion of STK10 via CRISPR/Cas9 and restoration of STK10 expression using lentivirus

The knockdown of STK10 in MV411 cells was achieved using the following gRNAs: Stk10 gRNA-1: CACCGACTCACCTCCAGCAGCTGCG; Stk10 gRNA-2: CACCGCATGATTGAGTTCTGTCCAG. Briefly, MV411 cells were transfected with a CRISPR/Cas9 vector (lentiCRISPR v2-P2A-PuroR) (Addgene, 52961) containing an STK10 guide RNA at a multiplicity of infection (MOI) of approximately 10 for 24 hours. Subsequently, the transfection was repeated for another 24 hours. Puromycin selection at a concentration of 2 μg/mL were then applied to isolate puromycin-resistant cells for a duration of 72 hours. Following selection, 50 individual cells were collected and seeded into separate wells of a 96-well plate to facilitate colony formation. These colonies were subsequently analyzed by immunoblot analysis to assess the expression levels of STK10.

The STK10-knockout cell line was infected with PLVX-tdTomato-STK10 at an MOI of 10. After 24 hours of infection, the supernatant containing excess lentivirus was removed by centrifugation, and the cells were then reinfected with the same virus for another 24 hours. Subsequently, the infected cells were collected, and the expression of STK10 was assessed using tdTomato detection via flow cytometry.

### ATP and HMGB1 release measurement

Supernatants from AML cell lines, including MV411, MOLM13, NOMO1, OCI-AML3/OA3, SHI1, THP1, and U937, as well as patient samples, were collected after 48 hours of treatment with SB633825 or Bortezomib alone or in combination. The ATP or HMBG1 content in these supernatants was then measured using the RealTimeGlo Extracellular ATP Assay or Lumit HMGB1 (Human/Mouse) Immunoassay (W6110) from Promega, following the manufacturer’s recommendations. The study was approved by the ethics committee of Ruijin Hospital, and all patients provided informed consent.

### ICD assessment *in vivo*

C57BL/6 mice were used for the C1498 xenograft model. Briefly, C1498 cells were exposed to a lethal dose of SB633825 and/or bortezomib. Subsequently, 2×10^6^ apoptotic C1498 cells were injected at Site 1, followed by 1×10^6^ viable C1498 cells at Site 2 one week later. The tumor volume at Site 2 was assessed after 10 days. The tumor volume was determined using the equation V = 1/2 × L × W^2, where V represents the calculated tumor volume. The study was conducted in accordance with the guidelines provided by the National Institutes of Health for the ethical treatment and utilization of animals in laboratory settings, and was approved by the ethics committee of Ruijin Hospital.

### Statistical analysis

The statistical analyses were conducted using GraphPad Prism 6 software. Paired or unpaired Student’s t-tests were employed to compare two experimental conditions. One-way or two-way ANOVA tests were utilized for comparing multiple conditions. Unpaired t-tests were used to assess differences in gene expression and CIBERSORT scores between C1 and C2 subtypes, provided that the normality assumption was met. Survival analyses were performed using the log-rank (Mantel-Cox) test.

## Results

### Two ICD-related clusters were identified with consensus clustering

**Figure 1:**
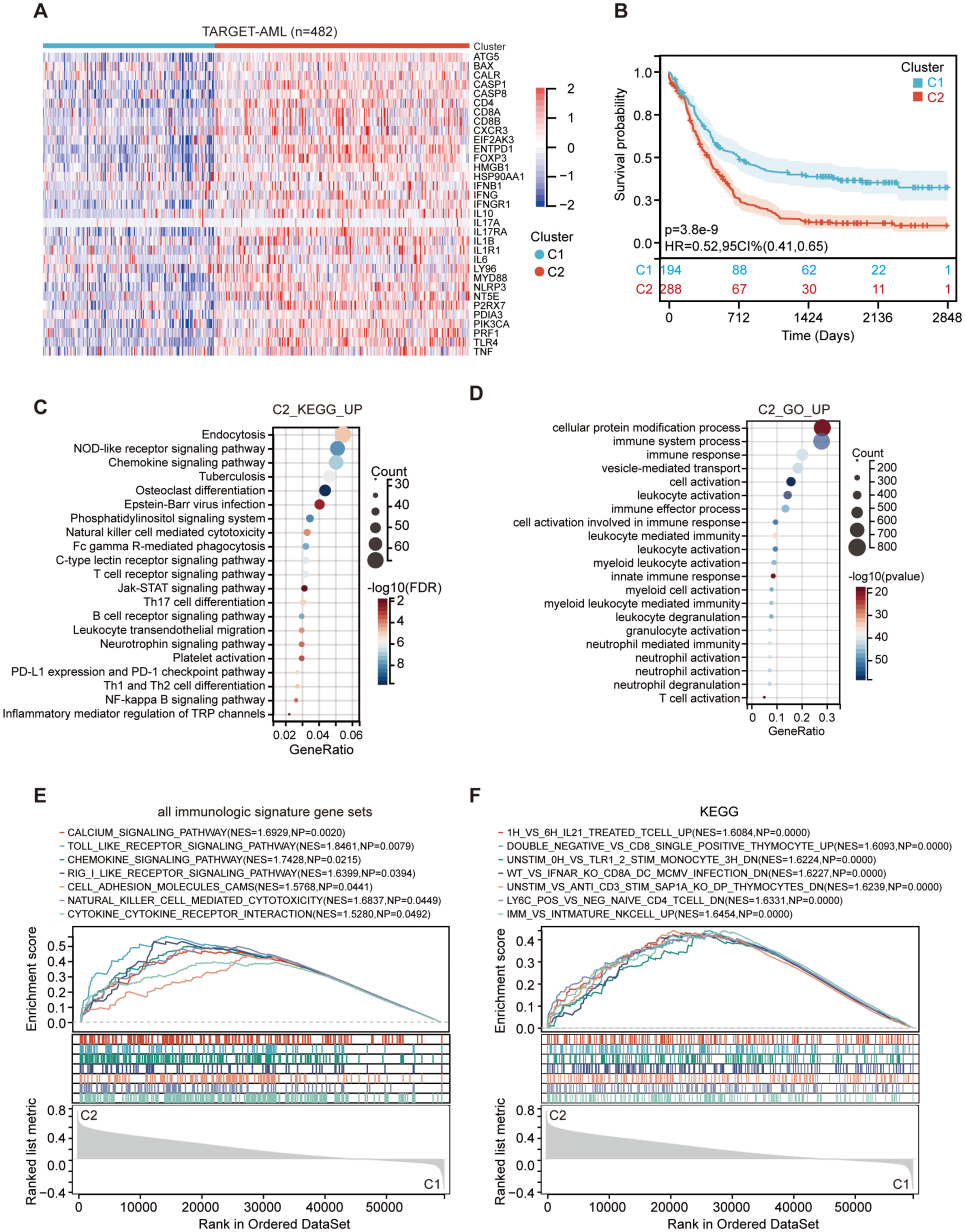
Identifying signaling pathways in the C1 (ICD-low subtype) and C2 (ICD-high subtype) subtypes within the TARGET dataset. **(A)** An interactive heatmap displays the expression of 33 ICD-related genes in the two subtypes, with red indicating high expression and green indicating low expression. **(B)** Kaplan-Meier curves for C1 (ICD-low subtype) and C2 (ICD-high subtype). The color patches represent the 95% confidence intervals. **(C, D)** The dot plot presents the enrichment analysis of KEGG **(C)** and GO **(D)** signaling pathways of C2 vs C1. The size of each dot corresponds to the gene count, while the color bar indicates -log10 (adjust p-value) or -log10 (FDR). **(E, F)** GSEA analysis determines the underlying signaling pathways between C1 and C2 subtypes.

An overview of the ICD-related genes was published by Abhishek et al. (23). To assess the impact of ICD on the progression of AML, we analyzed the expression profile of ICD-related genes in both normal and AML patient cohorts from the TARGET datasets. In contrast, nearly all ICD-related genes exhibited high expression levels in corresponding normal samples but were generally under-expressed in AML samples, with significant heterogeneity in their expression profiles (**Supplementary Figure 1A**). Based on consensus clustering, we identified two ICD-associated clusters in the TARGET AML cohort through k-means clustering (**Supplementary Figures 1B-D**). Cluster C1 exhibited low levels of expression of ICD-related genes, while cluster C2 displayed high levels of expression (**Figure 1A**). Therefore, the C1 cluster with significantly diminished ICD gene expression is designated as the ICD-low subtype. Conversely, the C2 clusters with markedly elevated ICD gene expression are referred to as the ICD-high subtype. However, KM analysis revealed that cluster C2, namely ICD-high subtype, had a shorter survival (**Figure 1B**).

Subsequently, a similar analysis of TCGA AML patients showed that GTEX normal blood samples had significantly elevated expression levels of ICD-related genes in comparison to TCGA AML samples (**Supplementary Figure 1E**). We also identified two clusters in the TCGA (**Supplementary Figure 1F**), GSE71014 (16) and GSE16432 (17) datasets (data not shown) by consensus clustering analysis. Notably, the ICD-high subtype also demonstrated a significantly reduced survival duration (**Supplementary Figures 2A-D**), consistent with observations in TARGET AML cohorts (**Figures 1A, B**). Finally, the two clusters of GSE16432 showed no significant difference in clinical outcomes (**Supplementary Figures 2E, F**). Overall, high expression of the ICD genes was positively associated with poorer patient outcomes in AML.

### ICD subtype-specific differentially expressed genes and signal pathways

Given that the two clusters within all AML cohorts showed different prognoses, we focused on identifying the key genes and signaling pathways that influence these outcomes. We identified 91 downregulated genes and 6,680 upregulated genes in the C2 subtype (**Supplementary Figures 3A, B**), with the upregulated genes showing enrichment in immunity-related activities, including leukocyte activation, myeloid cell activation, neutrophil activation, T cell activation, Th17 cell differentiation, Th1 and Th2 cell differentiation, and Natural killer cell-mediated cytotoxicity (**Figures 1C, D**). To further validate the prognostically relevant signaling pathways between the two subtypes, we conducted GSEA analysis and found that immune pathways, such as the chemokine signaling pathway and Toll-like receptor signaling pathway, were differentially enriched in the ICD-high subtype (**Figures 1E, F**). Our findings suggest that elevated expression of ICD genes in the subgroup with high ICD levels is indicative of an immune-activated microenvironment.

### Distinct tumor microenvironment landscape between two subgroups

**Figure 2:**
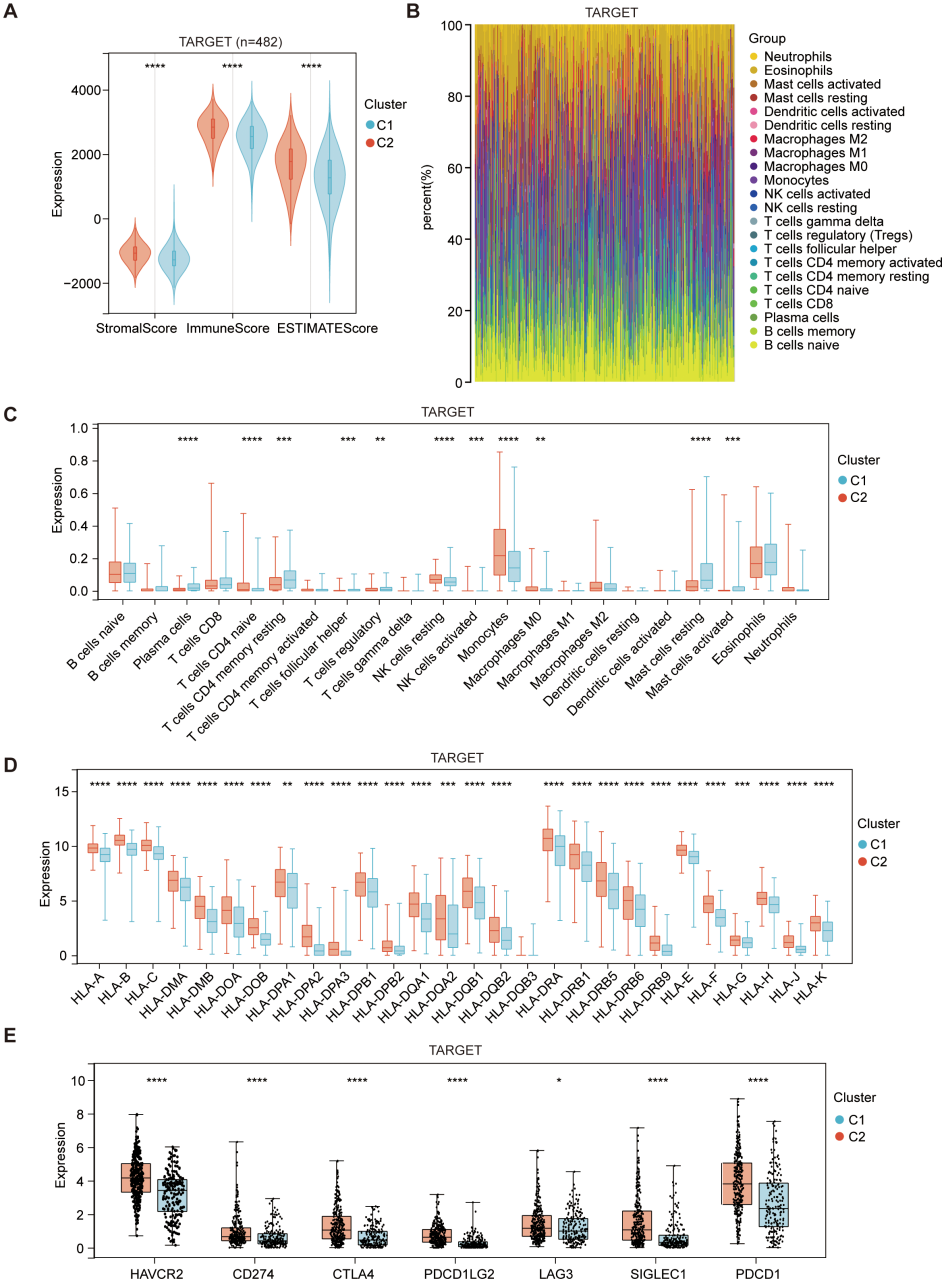
Immune composition of C1 (ICD-low subtype) and C2 (ICD-high subtype) subtypes in the TARGET dataset. **(A)** The violin plot presents the median and quartile estimations for stromal score, immune score, and ESTIMATE score. **(B)** The stack plots depict the relative proportions of 22 distinct immune cell types in each sample within the TARGET dataset. **(C)** The box diagram illustrates the relative distribution of distinct immune cell populations within the C1 and C2 subtypes. **(D, E)** Box plots depict differential expression of HLA genes **(D)**, and multiple immune checkpoints **(E)** between C1 and C2 subtypes. Statistical significance is denoted by *p < 0.05, **p < 0.01, ***p < 0.001, and ****p < 0.0001.

A favorable clinical prognosis is widely recognized as being associated with the abundance and activation of immune cells within the tumor microenvironment. To characterize the immune landscape of the two subtypes, we focused on analyzing the composition of the tumor microenvironment. Overall, the stromal, immune and estimate scores were higher in the C2 subtype compared to the C1 subtype in both cohorts (**Figure 2A**, **Supplementary Figure 4A**). The immune infiltration in AML microenvironment was assessed using the CIBERSORT approach along with LM22 signature matrix. The proportions of 22 immune cells, including both myeloid and lymphoid immune cells, were summarized in **Figure 2B** for the analysis of 482 AML patients from TARGET and in **Supplementary Figure 4B** for the analysis of 104 AML patients from GSE71014. Notably, there was a significant increase in monocytes, while mast cells showed substantial downregulation in the C2 subtype (**Figure 2C**, **Supplementary Figure 4C**). Additionally, changes in the number of monocytes and mast cells were validated between the two subtypes in GSE76009 (19) (**Supplementary Figure 4F**). In addition to the changes in immune cell composition, immune activation-associated molecules, such as human leukocyte antigen (HLA) genes and immune checkpoints, were also upregulated in the C2 subtype (**Figures 2D, E**, **Supplementary Figures 4D, E**).

These findings strongly suggest that the ICD-high subtype displays an activated immune phenotype, while the ICD-low subtype presents a suppressed immune phenotype in AML. This parallels the immunological characteristics observed in the majority of solid tumor microenvironments. However, the high expression of ICD genes in the immune-hot state correlates with a poorer prognosis in AML, marked by a notable increase in monocyte numbers and a significant decrease in mast cell numbers. The underlying cause of this peculiar phenomenon remains unclear, and the potential impact of alterations in monocyte and mast cell numbers on prognosis is yet to be determined.

### Highly similar STK10 expression profile and ICD gene expression profile

**Figure 3:**
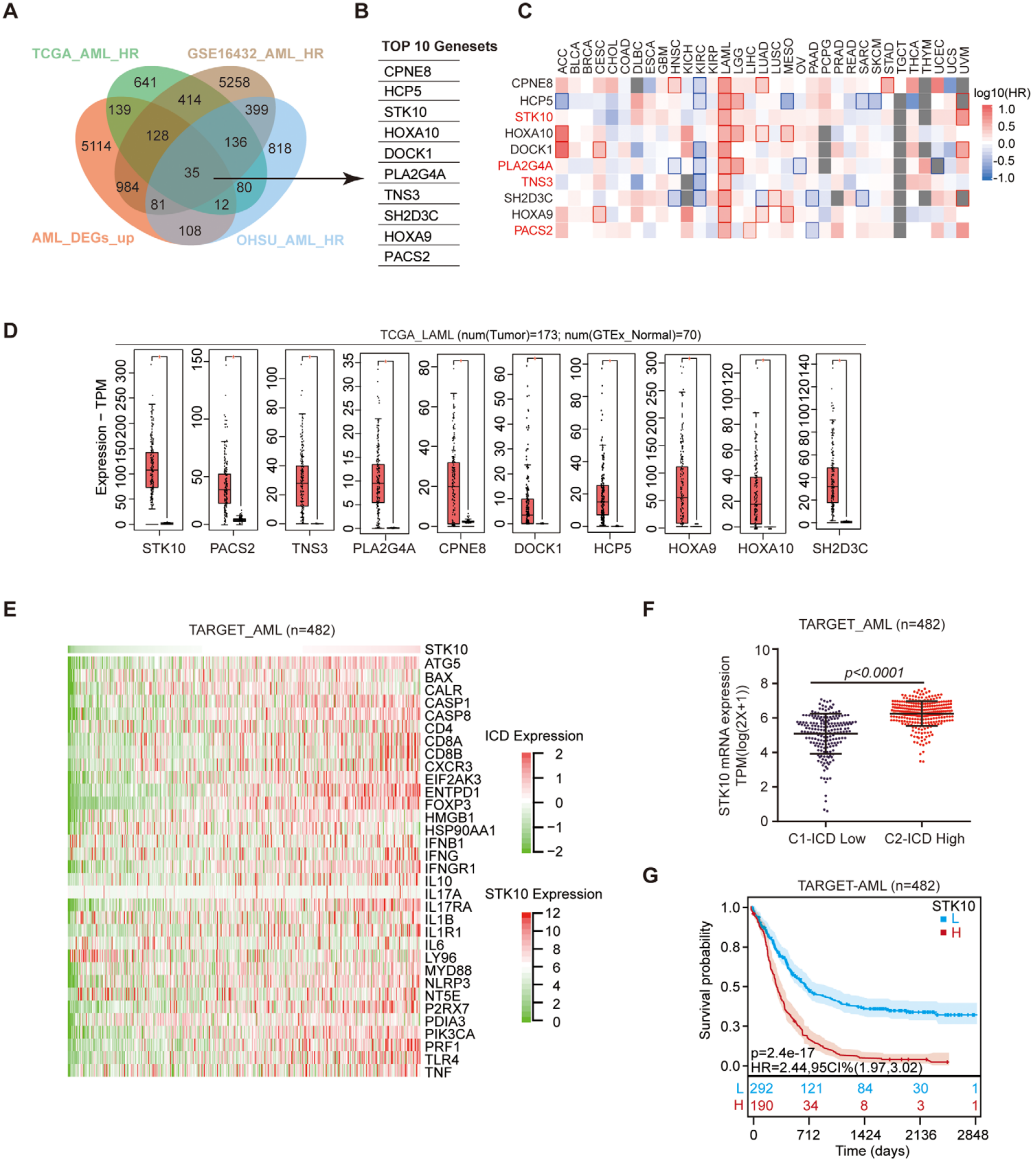
The expression of STK10 shows a positive correlation with the expression of ICD. **(A)** Wayne diagram illustrating the integration of TCGA AML HR, GSE16432 AML HR, OHSU AML HR, and TARGET AML upregulating DEGs datasets. **(B)** The intersection segment of the Wayne plot reveals a total of the top 10 genes that exhibit differential expression. **(C)** The heatmap displays the hazard ratio (HR) of 10 genes across 33 tumors. The red square with a red border indicates HR ≥ 1 and p ≤ 0.05, while the plain red square represents HR ≥ 1 and p > 0.05, indicating an association with poor prognosis. Conversely, the blue square signifies HR < 1, indicating a favorable prognosis. **(D)** The expression of 10 differentially expressed genes (DEGs) was examined in TGGA AML and GTEx normal samples. **(E)** The heat map visualizes the expression patterns of STK10 and 33 ICD genes in TARGET AML cohorts. **(F)** The scatter plot depicts the mRNA expression levels of STK10 in the ICD-high and ICD-low subtypes obtained from the TARGET dataset. **(G)** The prognosis of the TARGET cohort was evaluated using Kaplan-Meier analysis after stratifying the group into high and low subtypes based on STK10 expression.

To elucidate the perplexing phenomenon of heightened ICD gene expression and unfavorable prognosis, we extensively screened across diverse AML databases to investigate potential interactions between ICD genes and other pertinent prognostic factors (**Figure 3A**). This analysis revealed ten top-ranked risk genes that exhibited significant differences (Hazard Ratio ≥ 1 and p-value ≤ 0.05) (**Figure 3B**). In addition to their association with AML prognosis, CPNE8, HOXA10, and SPINT2 were also found to be prognostically relevant across multiple tumor types (**Figure 3C**).

Regarding specificity to AML, STK10, PLA2G4A, TNS3, and PACS2 demonstrated remarkable specificity (**Figure 3C**). In terms of expression levels, STK10 displayed the highest expression in AML, showing the most substantial fold difference (**Figure 3D**). This suggests the need for further investigation into STK10. The expression pattern of STK10 closely mirrored that of each ICD-related gene (**Figure 3E**, **Supplementary Figures 5A, B**), with its expression in the ICD-high subtype being significantly higher than that in the ICD-low subtype (**Figure 3F**). Moreover, high expression of STK10 predicted a poor prognosis, which aligned with the unfavorable prognosis observed in the ICD-high subtype (**Figure 3G**, **Supplementary Figure 5C**).

### STK10 inhibition leads to the modulation of ICD gene expression profile

**Figure 4:**
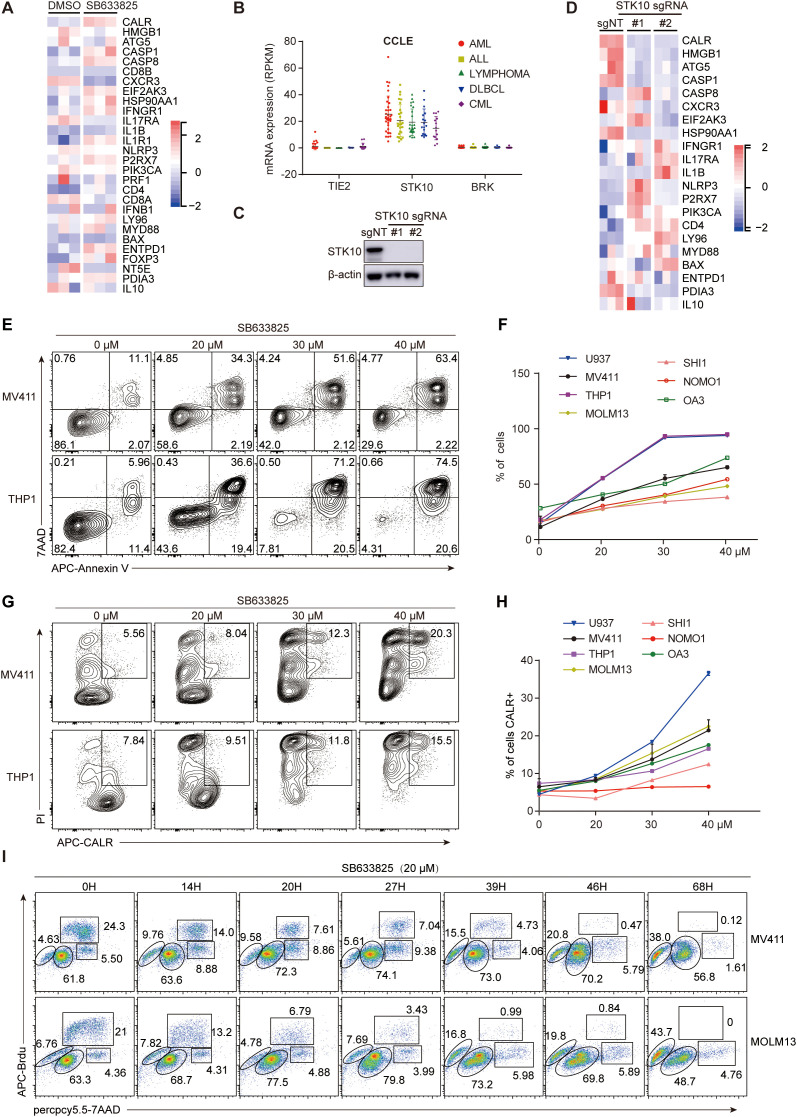
The inhibition of STK10 induces ICD and alters the expression profile of ICD-associated genes. **(A)** The heatmap illustrates the expression of ICD genes in MV411 cells, which were treated with SB633825 for 24 hours and underwent RNAseq analysis. **(B)** The expression levels of TIE2, STK10, and BRK mRNA were analyzed in various leukemia cell lines within the CCLE dataset (https://sites.broadinstitute.Org/ccle). **(C)** The STK10 gene was knocked out in MV411 cells using CRISPR-Cas9 technology. Two single monoclonal cells were selected for western blot analysis to detect the expression of STK10 protein after treatment with different lentiCRISPR-sgRNA. **(D)** The heatmap illustrates the expression of ICD genes in MV411 cells following the knockout of the STK10 gene. **(E–H)** The AML cells were treated with the STK10 inhibitor SB633825 at the indicated doses for 48 hours. Subsequently, they were subjected to flow cytometry analysis to measure apoptosis **(E, F)** and CALR exposure **(G, H)**. **(I)** The MV411 or MOLM13 cells were subjected to SB633825 treatment for varying durations, and then the distribution of cells in different phases of the cell cycle was assessed using BrdU/7AAD staining.

To investigate whether STK10 regulates ICD gene expression or influences ICD-induced immune activation, we treated AML cell lines with SB633825 or knocked out the STK10 gene to assess its impact on cell viability and CALR extracellular exposure. The compound SB633825 exhibits potent inhibitory activity against TIE2, STK10, and BRK by competing with ATP binding. However, considering the significantly low expression levels of TIE2 and BRK in AML cells (**Figure 4B**), SB633825 can be regarded as a selective inhibitor specifically targeting STK10 in AML. The RNAseq results revealed that treatment with SB633825 led to the up-regulation of the majority of ICD-related genes (**Figure 4A**). The STK10 gene was successfully disrupted using CRISPR-Cas9 (**Figure 4C**), leading to a significant down-regulation of key components involved in ICD, such as CALR, HMGB1, ATG5, and CASP1 (**Figure 4D**). Additionally, an escalated dose of SB633825 induced apoptosis (**Figures 4E, F**) and enhanced CALR exposure on the cell membrane in a dose-dependent manner (**Figures 4G, H**). Mechanistically, SB633825 arrested the cell cycle at the G0/G1 phase, ultimately leading to apoptosis (**Figure 4I**).

**Figure 5:**
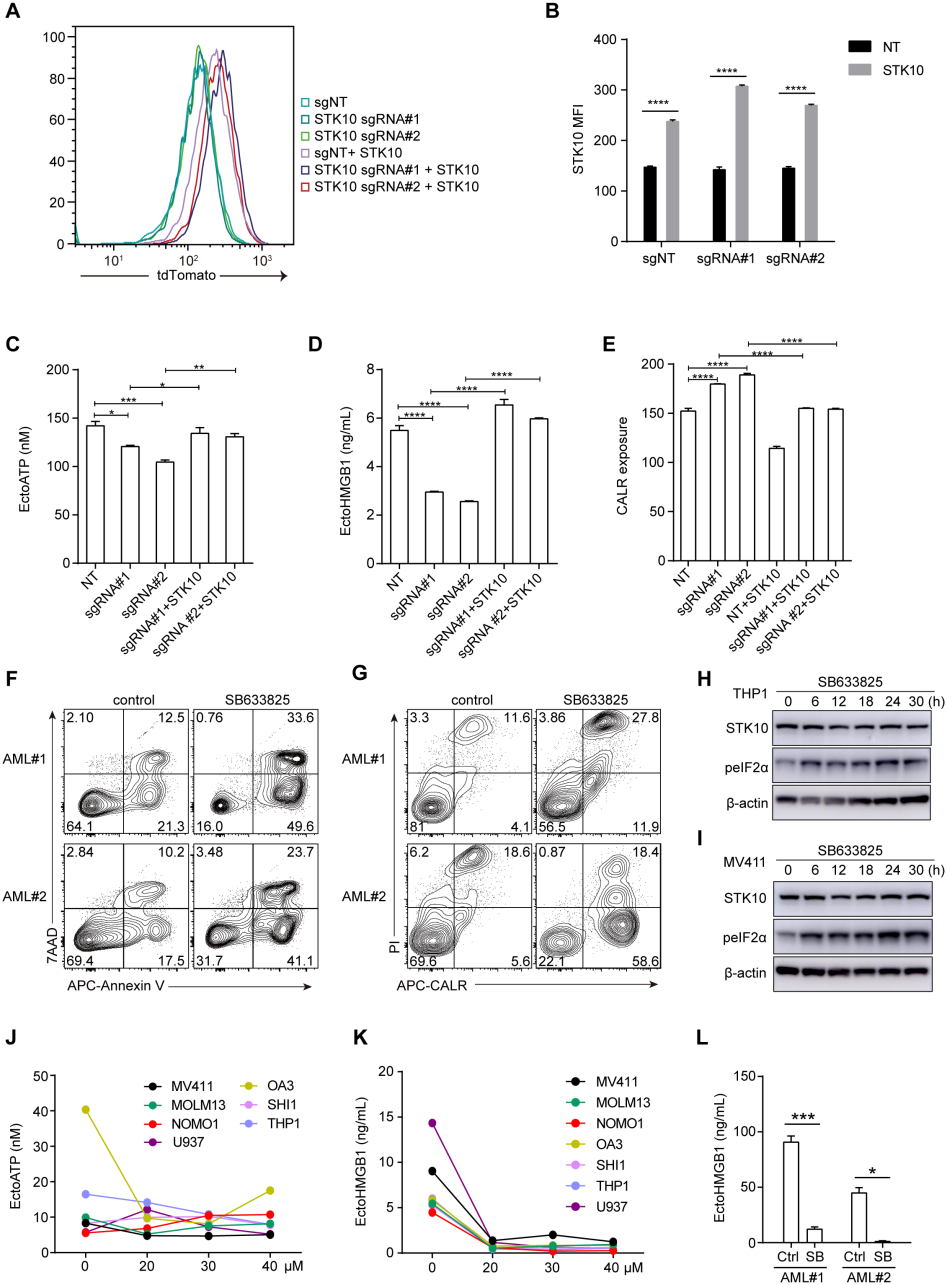
The rescue of STK10 inhibits CALR exposure, while the inhibition of STK10 also triggers ICD in AML patients. **(A)** The expression of RFP-STK10 was detected by flow cytometry (FCS) after rescuing the knockout of the STK10 gene using CrisprCas9 in MV411 cells. **(B)** The bar chart represents the MFI statistics for RFP-STK10. **(C–E)** Detect the release of ATP **(C)**, HMGB1 **(D)**, and exposure of CALR **(E)** following STK10 rescue in MV411 cells with CRISPR-Cas9-mediated knockout of the STK10 gene. **(F, G)** The two AML samples were treated with a concentration of 30 μM SB633825 for 48 hours, followed by assessment of apoptosis **(F)** and CALR exposure **(G)** using FCS. **(H, I)** THP1 **(H)** and MV411 **(I)** cells were treated with 30 μM SB633825 for various durations, followed by detecting STK10 expression and phosphorylated eIF2α levels through western blot analysis. **(J)** The extracellular ATP accumulation in different AML cells was evaluated following a 48-hour treatment with SB633825. **(K)** Release of HMGB1 in the cell supernatant in response to the indicated dose of SB633825 treatment on AML cells for 48 hours. **(L)** Two AML patient samples were treated with 30 μM SB633825 for 48 hours, and the release of HMGB1 into the culture supernatant was assessed. Statistical significance is indicated by *p < 0.05, **p < 0.01, ***p < 0.001, and ****p < 0.0001.

To further clarify the relationship between STK10 and ICD-related genes, we employed a tdTomato-STK10 lentivirus to restore the knocked-out STK10, and the restoration of STK10 was successful (**Figures 5A, B**). Upon genetic knockout of STK10, there was a marked reduction in the release of ATP and HMGB1, accompanied by a significant increase in CALR exposure (**Figures 5C-E**). Following the rescue of STK10, the release of ATP and HMGB1 was reinstated, while the upregulation of CALR exposure was notably diminished (**Figures 5C-E**).

Furthermore, SB633825 also significantly induced tumor cell apoptosis and CALR exposure in two AML patients (**Figures 5F, G**). In addition to CALR exposure, the release of HMGB1, ATP, and phosphorylation of eIF2α also play a pivotal role in eliciting an immune response during ICD. Treatment with sublethal concentrations of SB633825 led to increased phosphorylation of eIF2α in a time-dependent manner (**Figures 5H, I**), with negligible effects on ATP release across multiple AML cell lines (**Figure 5J**). Nonetheless, the secretion of HMGB1 was attenuated in both primary AML patient samples and AML cell lines (**Figures 5K, L**). These findings align with those observed in AML cell lines, suggesting that each factor plays a distinct role in the process of ICD and demonstrates varying degrees of relevance to prognosis.

### Co-treatment with SB633825 and ICD inducers synergistically elicit antitumor effects

**Figure 6:**
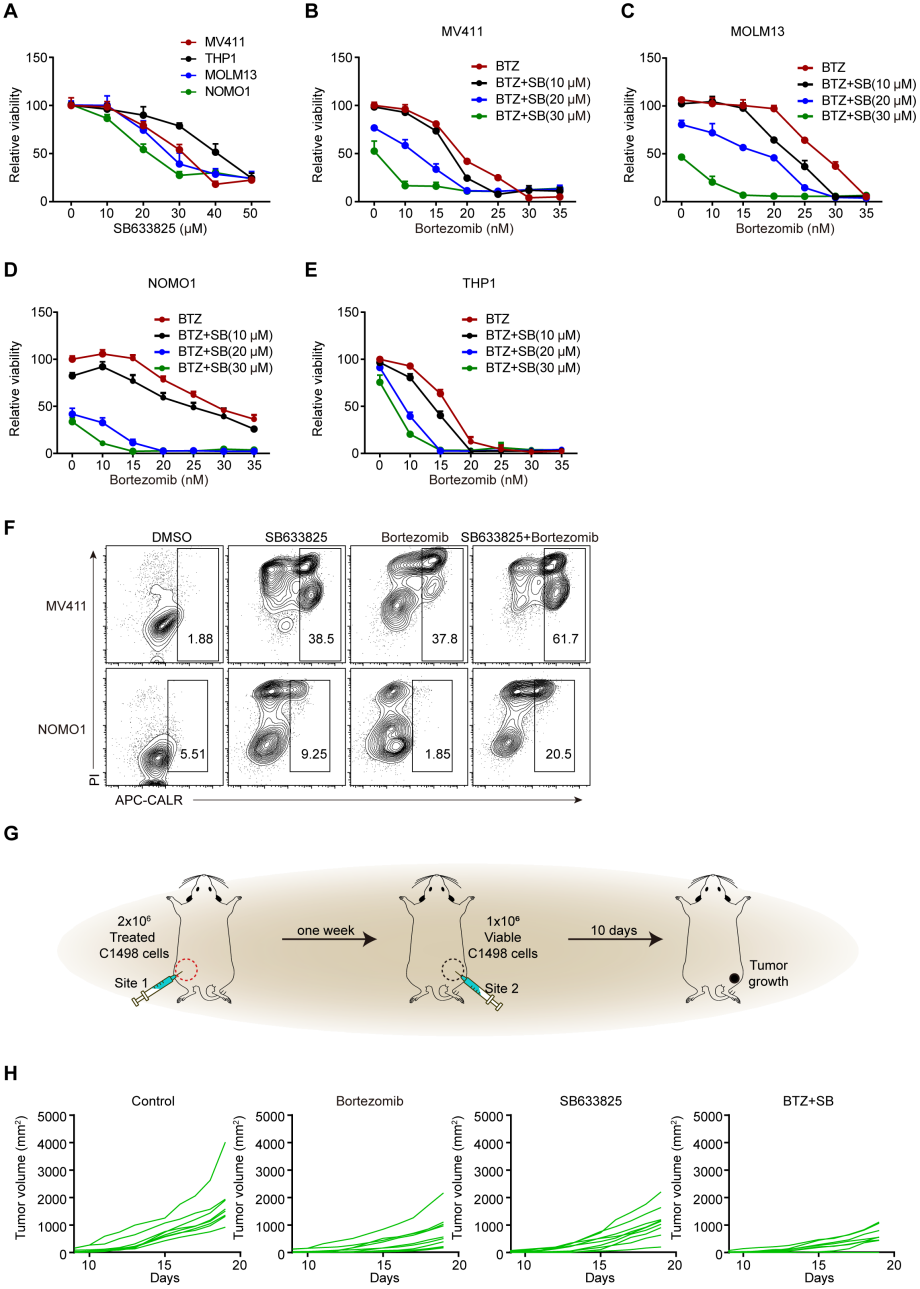
The combination of SB633825 with other ICD inducers enhances SB633825-induced ICD by promoting apoptotic cell death. **(A)** Relative viability was measured after treatment with increasing doses of SB633825 in AML cells. **(B–E)** Relative viability was measured after 48 hours of treatment with Bortezomib and increasing doses of SB633825 in MV411 **(B)**, MOLM13 **(C)**, NOMO1 **(D)**, and THP1 **(E)** cells. **(F)** Ecto-CALR exposure on MV411 and NOMO1 cells was assessed by FCS after 48 hours of treatment with SB633825 and Bortezomib individually, as well as in combination. **(G)** The mouse model for detection of SB633825-induced ICD *in vivo*. **(H)** The volume curve illustrates the changes in tumor volume in Site 2 upon treatment of C1498 cells with SB633825 and Bortezomib alone or in combination, injected into Site 1.

Previous studies have reported that bortezomib effectively induces ICD in AML cells (24). Whether there is an interaction between SB633825 and ICD inducers remains unclear. Concentration-dependent induction of apoptosis was observed in AML cell lines treated with SB633825 alone (**Figure 6A**). Meanwhile, co-treatment with bortezomib synergistically induced apoptosis and extracellular CALR exposure in AML cells (**Figures 6B-F**). The mouse leukemia cell line C1498 was subjected to treatment with SB633825 and Bortezomib alone or in combination. Subsequently, the treated C1498 cells were injected at site 1, followed by the injection of untreated C1498 cells at site 2 after a week, and then the growth of tumors was monitored (**Figure 6G**). The antitumor effects of both SB633825 and bortezomib alone were observed as anticipated, but their combination demonstrated a synergistic enhancement of the antitumor immune response (**Figure 6H**).

**Figure 7:**
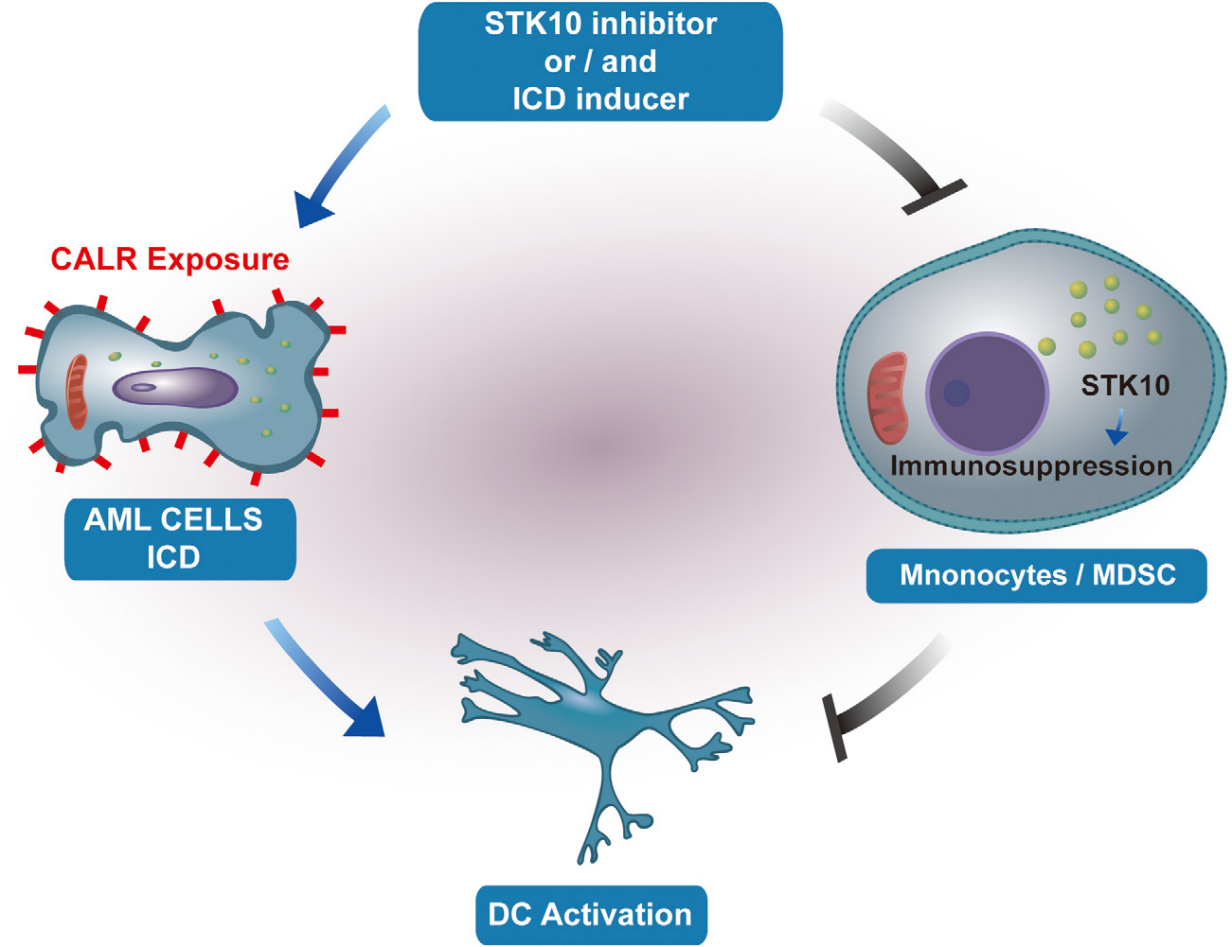
The illustration portrays the impact of STK10 on the occurrence of ICD and the function of monocytes/MDSCs.

Consequently, our hypothesis posits that STK10 does not exhibit a direct regulatory association with the elevated expression of immunogenic cell death (ICD) genes. While the upregulation of ICD responses within the tumor microenvironment further stimulates the immune system—such as by promoting dendritic cell (DC) maturation and antigen presentation—STK10 concurrently activates inhibitory immune cells, including myeloid-derived suppressor cells (MDSCs) and M2 macrophages, which counteract the immune activation induced by ICD upregulation. The proposed mechanism, illustrated in **Figure 7**, indicates that this mode of action may be applicable to various types of tumors.

## Discussion

An in-depth analysis of gene expression patterns in AML samples has unveiled further insights into the heterogeneity of this disease. In addition to stratifying AML based on high and low ICD subtypes according to gene expression profiles, we have identified distinct molecular signatures associated with each subtype. Our research has unveiled a significant link between elevated expression levels of ICD genes and a relatively unfavorable prognosis in AML patients, a correlation that has also been noted in specific types of solid tumors (25–29). This contradicts previous reports that indicated increased expression of these genes is linked to better overall survival rates. These unexpected results underscore the complexity of AML biology and emphasize the necessity for further investigation into the underlying mechanisms propelling disease progression.

Accordingly, we have also delved into the cellular components within AML samples. Our data reveal a higher proportion of monocytes and mast cells in the ICD high subtype, raising intriguing questions about potential interactions between these cell types and their impact on disease prognosis. The release of tumor protein, translationally-controlled 1 (TPT1) by dying cancer cells has been shown to facilitate the recruitment of myeloid-derived suppressor cells (MDSCs) to the tumor microenvironment (TME), thereby promoting local immunosuppression and disease progression (30, 31). The classification of monocytes, particularly those with high proportions in ICD-high subtype, as MDSCs is currently uncertain. It is hypothesized that the significant decline in overall survival might not be due to the upregulation of the ICD gene, but rather by the accumulation of monocytes or MDSCs. There is a potential for monocytes and mast cells to impact the tumor microenvironment in AML. However, verifying this hypothesis through direct methods is currently unfeasible due to difficulties in patient sample collection and overall survival evaluation.

Although not all cell-death inducers can activate intracellular stress pathways, considerable efforts have been dedicated to deciphering the release of damage-associated molecular patterns (DAMPs) by malignant cells in response to immunogenic stressors such as bortezomib and crizotinib (11, 32, 33). In this study, we explored the role of STK10 in shaping the tumor microenvironment, given its significant correlation with genes associated with ICD expression in both TARGET and GEO cohorts. Treatment with SB633825 in AML cell lines and samples results in minimal passive release of HMGB1, heightened exposure of CALR, and increased phosphorylation of eIF2α upon apoptosis induction at a half-lethal dose of SB633825. The inhibition of STK10 contributes to the induction of ICD, suggesting a potential indirect regulatory link between STK10 and ICD genes. It is plausible that STK10 modulates immune activation through direct regulation of other immune cells, such as monocytes, MDSCs or mast cells.

Accumulating evidence shows that BTZ triggers ICD through upregulating type I interferon (IFN) signaling (11, 34). Our findings suggest that the STK10 inhibitor SB633825, in collaboration with bortezomib, enhances CALR exposure, thereby demonstrating a more robust anti-tumor immune activation. However, the impact of SB633825 on type I IFN signaling remains unclear. Additional research is warranted to investigate the precise mechanism by which SB633825 activates intracellular stress pathways.

The cascade of events in the progression of ICD can lead to significant infiltration of myeloid and lymphoid cells, shifting neoplastic lesions from a ‘cold’ phenotype to a ‘hot’ phenotype (3). However, malignant cells employ diverse strategies to evade immune surveillance by circumventing the emission or detection of ICD-relevant signals (35). Further investigation is required to fully comprehend STK10’s specific role in immune surveillance. Although its exact function remains unclear, our study lays a solid foundation for future research on how STK10 contributes to tumor immunity.

## Data Availability

The datasets presented in this study can be found in online repositories. The names of the repository/repositories and accession number(s) can be found below: https://www.ncbi.nlm.nih.gov/, GSE16432,GSE71014,GSE76009.
