# Supplementary figures and images for: The enhancement of immunoactivity induced by immunogenic cell death through serine/threonine kinase 10 inhibition: a potential therapeutic strategy
Source: Front Immunol. 2024 Nov 1;15:1451796. doi: 10.3389/fimmu.2024.1451796 (PMC11563836; doi:10.3389/fimmu.2024.1451796)

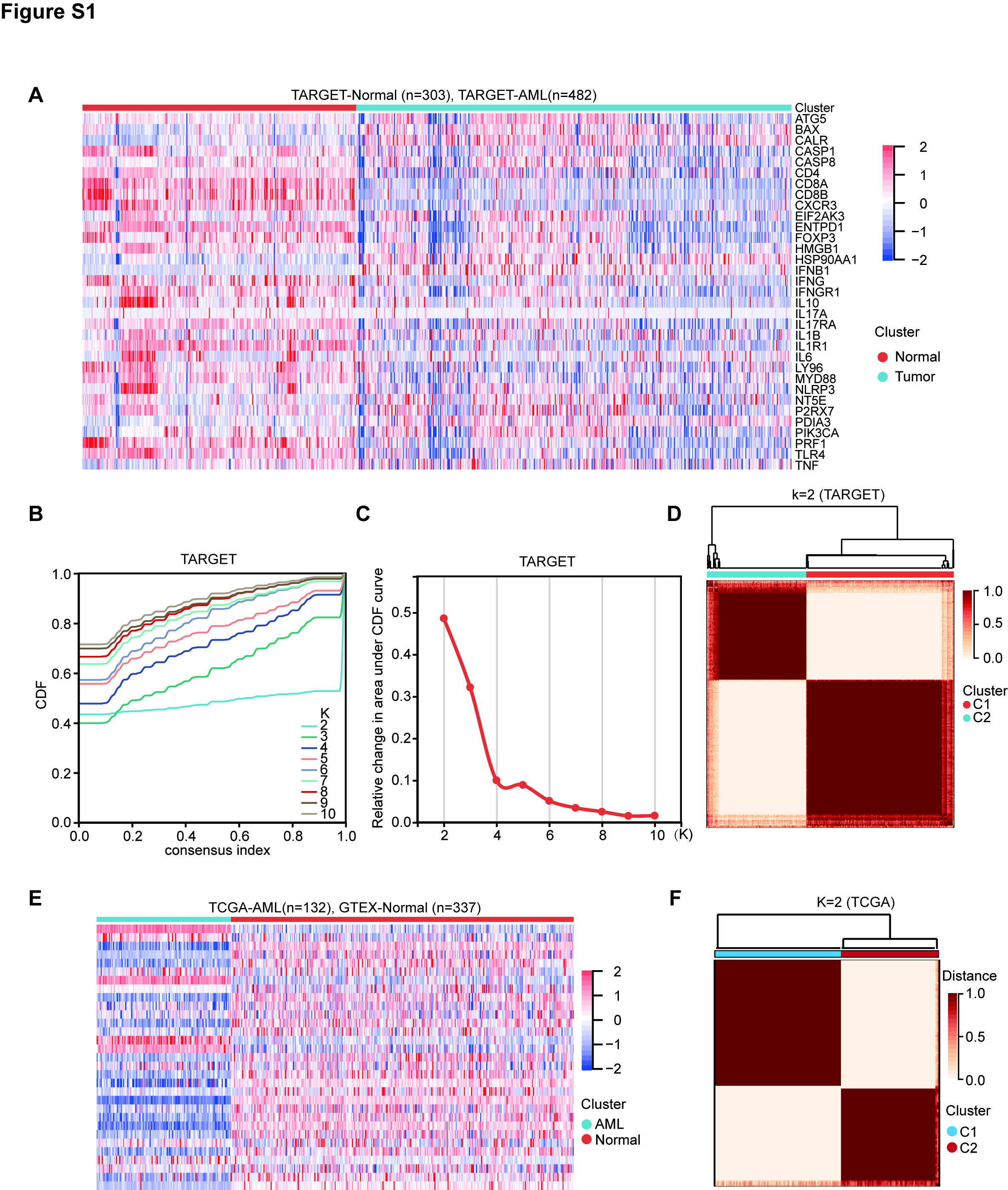

Supplement: Supplementary file 1 [file Image1.tif]

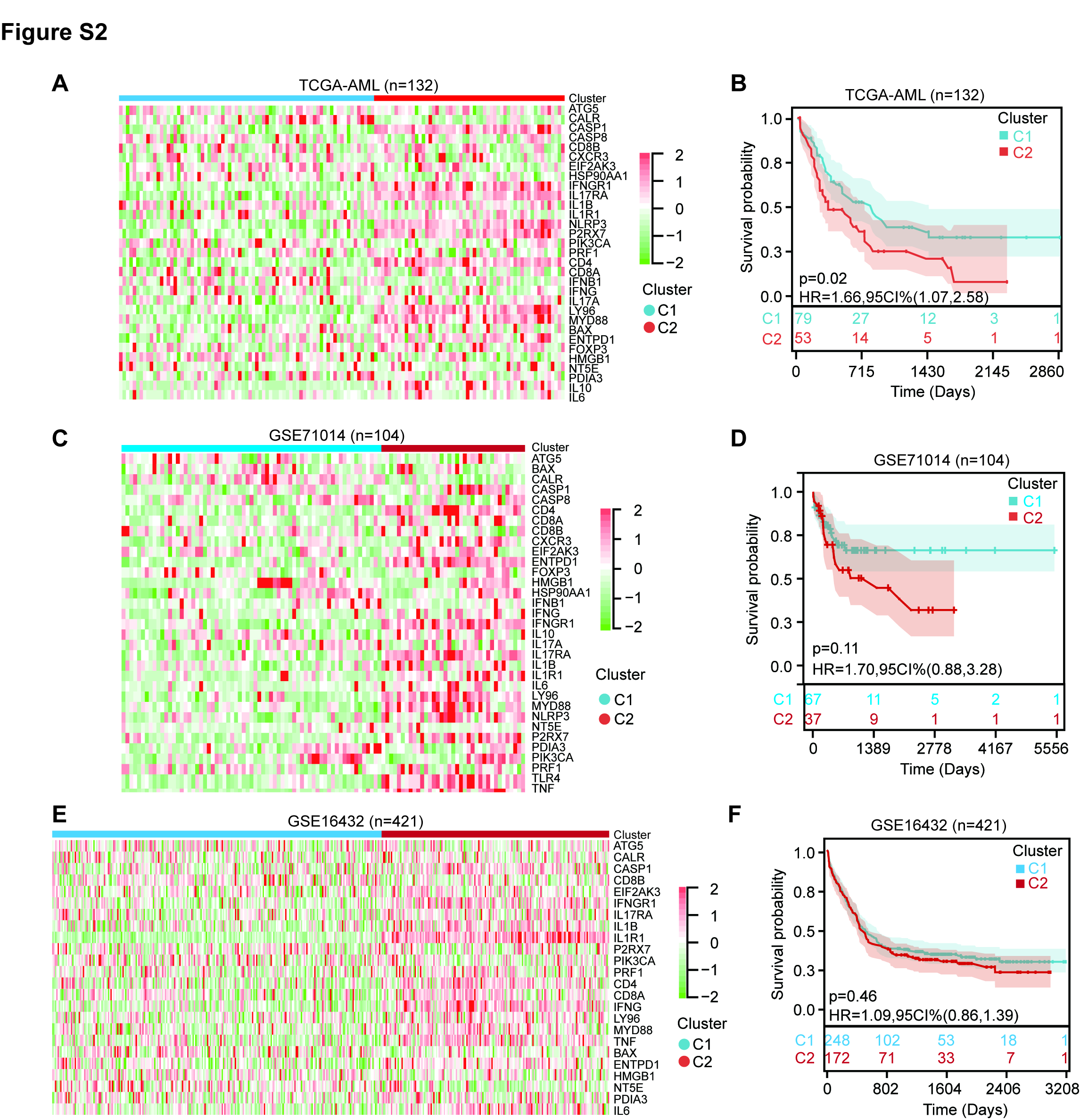

Supplement: Supplementary file 2 [file Image2.tif]

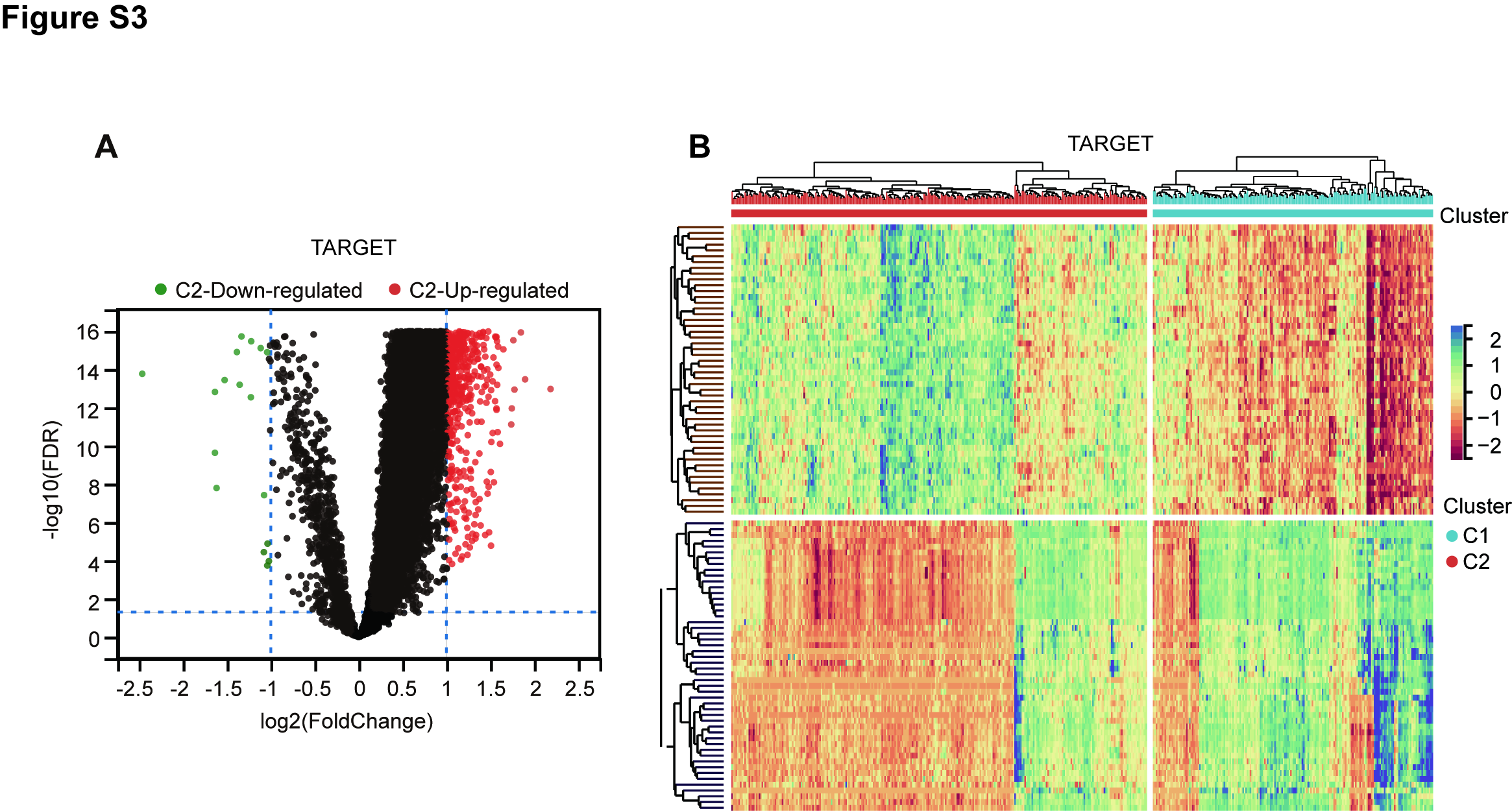

Supplement: Supplementary file 3 [file Image3.tif]

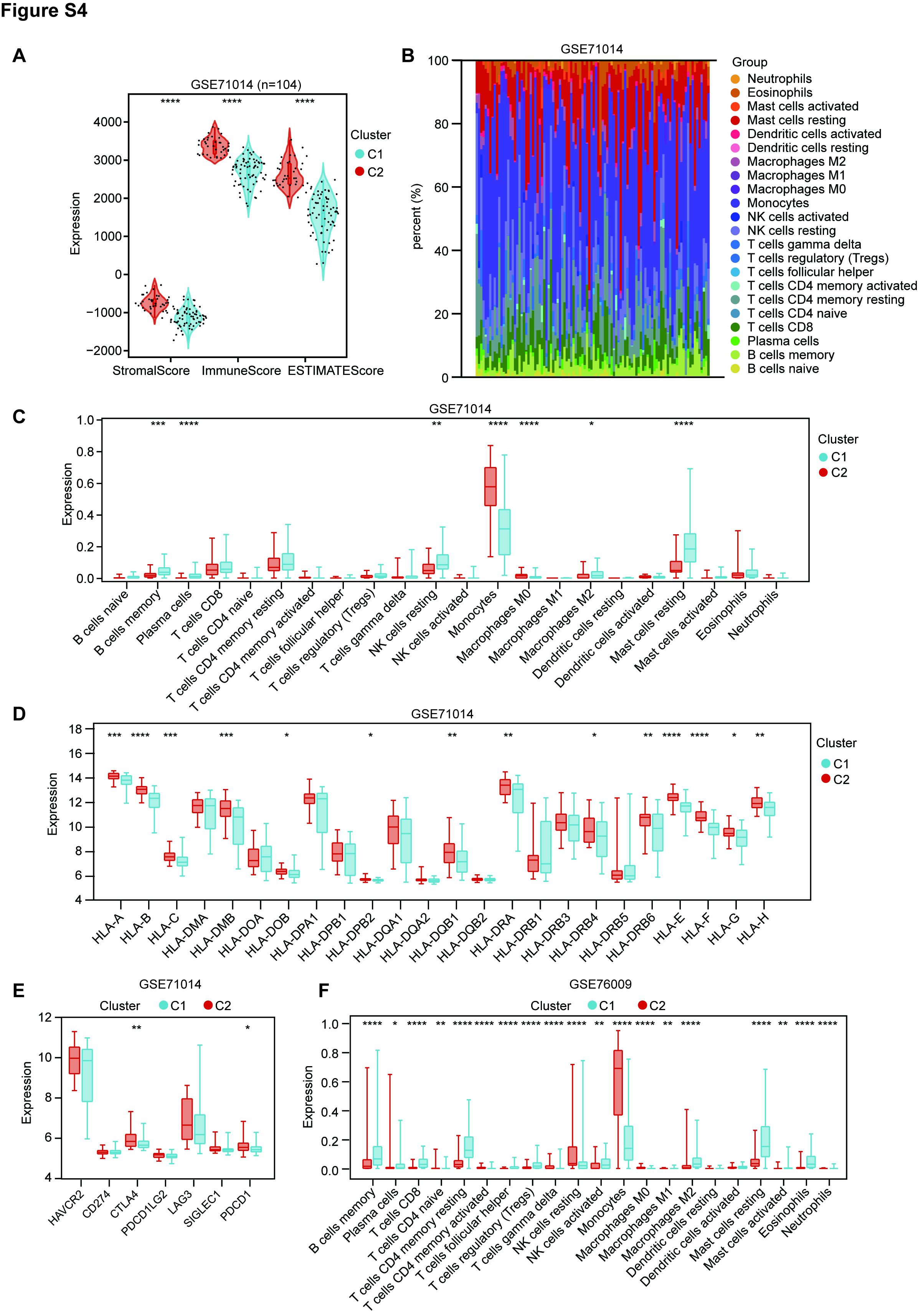

Supplement: Supplementary file 4 [file Image4.tif]

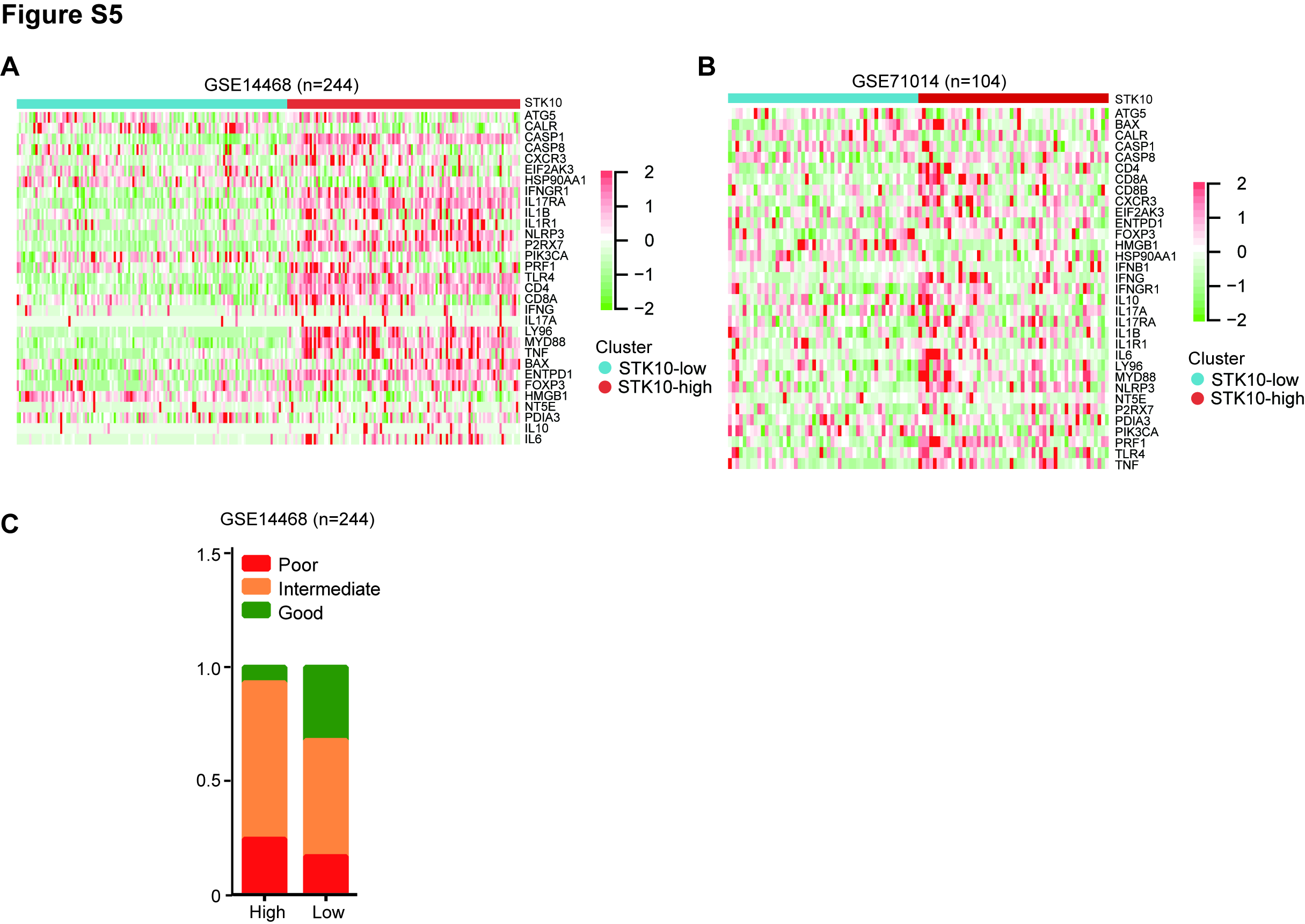

Supplement: Supplementary file 5 [file Image5.tif]
